# Irie Classroom Toolbox: a study protocol for a cluster-randomised trial of a universal violence prevention programme in Jamaican preschools

**DOI:** 10.1136/bmjopen-2016-012166

**Published:** 2016-05-10

**Authors:** Helen Baker-Henningham, Marcos Vera-Hernández, Harold Alderman, Susan Walker

**Affiliations:** 1 School of Psychology, Bangor University, Bangor, Gwynedd, UK; 2 Tropical Medicine Research Institute, University of the West Indies, Mona, Kingston, Jamaica; 3 Centre for the Evaluation of Development Policies, Institute of Fiscal Studies, London, UK; 4 International Food Policy Research Institute, Washington DC, USA

**Keywords:** violence prevention, low and middle-income countries, preschool, cluster randomised trial

## Abstract

**Introduction:**

We aim to determine the effectiveness of a school-based violence prevention programme implemented in Jamaican preschools, on reducing the levels of aggression among children at school, and violence against children by teachers.

**Methods and analysis:**

This is a 2-arm, single-blind, cluster-randomised controlled trial with parallel assignment. Clusters are 76 preschools in Kingston, and all teachers and classrooms in the selected schools are included in the study. In addition, a random sample of up to 12 children in the 4-year-old classes have been selected for evaluation of child-level outcomes. The intervention involves training teachers in classroom behaviour management and in strategies to promote children's social-emotional competence. Training is delivered through five full-day workshops, monthly in-class coaching over 2 school terms, and weekly text messages. The primary outcome measures are: (1) observed levels of child aggression and (2) observed violence against children by teachers. Secondary outcomes include observations of the levels of children's prosocial behaviour and the quality of the classroom environment, teachers’ reports of their mental health, teacher-reported child mental health, direct tests of children's self-regulation and child attendance.

**Ethics and dissemination:**

If this intervention were effective at improving the caregiving environment of young children in school, this would have significant implications for the prevention of child mental health problems, and prevention of violence against children in low and middle-income countries where services are often limited. The intervention is integrated into the school system and involves training existing staff, and thus, represents an appropriate strategy for large-scale implementation and benefits at the population level. Ethical consent for the study was given by the School of Psychology Ethics and Research Committee, Bangor University (ref: 2014-14167), and by the University of the West Indies Ethics Committee (ref: ECP 50,14/15).

**Trial registration number:**

ISRCTN11968472; Pre-results.

## Introduction

Violence is a leading worldwide public health problem, and makes a substantial contribution to the burden of disease at the global level.^1^ Interventions in early childhood are an important component in the primary prevention of violence. Training young children's caregivers in behavioural strategies to reduce child aggression and promote child social skills can (1) prevent the early development of antisocial behaviour and (2) reduce violence against children by caregivers.

Children with social, emotional and behavioural problems at school entry are at risk for many negative outcomes throughout life including juvenile delinquency, academic underachievement and crime and violence in adulthood.^2^ Schools offer a logical public health setting for implementing preventative interventions, and reviews of school-based violence prevention programmes in high-income countries indicate significant reductions to children's aggressive behaviour and increases in child competencies,^3^ with some evidence that benefits are sustained into the late teenage and adult years.^4^ However, there is limited evidence of the effectiveness of school-based preventative interventions in low and middle-income countries (LMIC) where schools often have poor physical conditions, high child-staff ratios, few classroom resources and low levels of teacher training.

School-based violence prevention programmes can also reduce the use of violence against children by teachers.^5^ This is important for many LMIC where corporal punishment is a commonly used behaviour management strategy; though the evidence for effective teacher training interventions in LMIC is limited.^6^ A recent study demonstrated a reduction in physical violence by school staff against primary school children in Uganda.^7^ Exposure to corporal punishment in schools is associated with poor child mental health, poor social skills, increased aggression, low levels of school achievement and increased school drop-outs.^6^

Child mental health services are severely limited in LMIC,^8^ and there is a growing interest in integrating these services into educational settings. If treatment services are made available in schools, it will be important that the schools are also engaged in the primary prevention of child mental health problems and are providing a supportive environment for child mental health promotion.

### The Jamaican context

Violence prevention is a national and regional priority for Jamaica and the wider Caribbean.^9^ A survey of a small, but nationally representative sample of Jamaican 5–6-year-old children reported a prevalence of externalising disorders of 12%, and none of these children had received any form of medical or social services.^10^ The prevalence is likely to be substantially higher in low-income, inner-city areas.^11^ In an efficacy trial, we screened 1733 children from 24 preschools in disadvantaged areas of Kingston using teacher reports, and 21% had four or more symptoms of conduct problems.^12^

Corporal punishment is also widespread in Jamaica and although banned by law in early childhood institutions, it continues to be widely used.^5^ Corporal punishment by teachers is associated with poor school achievement among Jamaican primary school children.^13^ In focus groups, although parents and teachers of preschool children reported using corporal punishment to manage child behaviour, they also reported that it is undesirable and ineffective.^14^ Jamaica is an ideal location for integrating a violence prevention programme into preschools due to the well-established preschool network, with over 97% of 3–5-year-old children enrolled in school.

### Development of the Irie Classroom Toolbox

The intervention to be used in this study has been developed from our experiences implementing and evaluating behavioural interventions to prevent violence and promote child mental health in Jamaican preschools. Our first step was to conduct formative research followed by a pilot study in five preschools in which we trained teachers in classroom behaviour management, and the research team delivered a 14-session curriculum unit on social and emotional skills in each intervention classroom. Teachers were trained through seven full-day workshops, and were provided with extensive in-class support (1 h/week for 6 months) and a monthly, individual consultation session. The intervention was based on the Incredible Years Teacher Training Programme,^15^—an evidence-based programme to prevent and treat child conduct problems, although we made some modifications to facilitate use with teachers with limited prior training. A qualitative and quantitative evaluation of this pilot study was conducted, and we found large benefits to teacher practices and classroom atmosphere (mean effect size (ES)=2.15),^5^ and significant benefits to teacher-reported child mental health (mean ES=0.38).^16^ The qualitative evaluation highlighted the aspects of the intervention teachers found most useful, and why, what strategies were misunderstood, and teachers also provided suggestions for improving the intervention.^17^ The results of the pilot study were used to prepare additional training materials to make it more suitable for the Jamaican preschool context. These training materials included producing Jamaican video vignettes, developing hands-on activities, role-plays and group work to introduce key principles of behaviour management, and adding training content to address teachers’ unmet needs. We also developed a curriculum to teach social-emotional skills that could be integrated into ongoing teaching and learning activities, rather than using a separate curriculum unit.

We implemented and evaluated this modified intervention in an efficacy trial in 24 preschools.^12^ Teachers attended eight full-day workshops and received 1 hour of in-class support each month over 4 months. A quantitative and qualitative evaluation was again conducted, and benefits to teacher practices and classroom atmosphere were of a similar magnitude to those found in the pilot study (mean ES=2.5), despite the large reduction in in-class support. The intervention also produced meaningful benefits to high-risk children's conduct problems and prosocial behaviour at home and at school with a mean ES of 0.49 SD.^12^ The qualitative evaluation focused on what aspects of the training were most useful and least useful, and factors affecting teachers’ implementation of the strategies.

We have developed ‘The Irie Classroom Toolbox using the results of the efficacy trial and our ongoing documentation of training Jamaican preschool teachers. Observational data of teachers’ use of strategies combined with teachers’ and facilitators’ reports and ratings of strategies which are easy to use, most effective and most relevant have been used to identify the key evidence-based behaviour principles that are most suitable and effective. In addition, teachers’ and facilitators’ reports of preferred training methods have been used to develop the training materials and to develop a protocol for the provision of in-class support. The modified intervention is based on the concept of a ‘toolbox’ consisting of ‘evidence-based kernels and behavioural vaccines’,^18^ that are used across a range of evidence-based child behaviour modification interventions, and uses evidence-based behaviour change techniques in the training process.^19^ This low-cost training package is designed to be suitable for use with teachers with limited training and working in poorly resourced settings in LMIC, and aims to facilitate widespread adoption of key child behaviour management strategies.

### Aims

The aims of this trial are to evaluate the effect of a universal violence prevention programme implemented in inner-city preschools in Kingston, Jamaica. The intervention involves training teachers in classroom behaviour management and in strategies to promote child social-emotional competence. The primary aims of the study are to reduce teachers’ use of violence against children and to reduce aggression among children. Additional aims are to improve the quality of the classroom environment, promote child social skills, self-regulation, mental health and school attendance, and to promote teachers’ mental health. The cost-effectiveness of the intervention will also be evaluated.

## Methods and analysis

### Design

The trial is a 2-arm, single-blind, cluster-randomised trial with parallel assignment. Clusters are community preschools catering to children aged 3–6 years in Kingston, Jamaica. Inclusion criteria are that preschools are situated in urban areas of Kingston, have 2–4 classrooms, and an average of at least 10 children per class. A random sample of 76 preschools were selected from all eligible schools. All teachers in the selected preschools are participating in the study, and a random sample of children in the 4-year-old class of each preschool was selected to participate in the evaluation. Children were eligible to participate in the evaluation aspect of the study if their average attendance in the preceding two terms was greater than 50%, and they have no obvious disability. However, all children within the school receive the intervention.

### Intervention

#### Content of training

The intervention involves training preschool teachers using ‘The Irie Classroom Toolbox’. The toolbox consists of four modules: (1) creating an emotionally supportive classroom environment, (2) preventing and managing child behaviour problems, (3) teaching social and emotional skills to children, (4) individual and class-wide behaviour planning. The first two modules consist of training in a number of discreet strategies which are feasible, relevant and flexible, and have readily observable effects (eg, verbal praise, use of visual cues). Each behaviour kernel can be used alone or in conjunction with others to modify child behaviour. This is a pragmatic approach that allows teachers to choose which strategies to use and how to implement them depending on their own teaching style, their classroom context and the needs of the children in their class. The third module provides teachers with lesson plans, visual aids and classroom activities to integrate the teaching of social-emotional competence into ongoing teaching and learning activities.

#### Process used in training

The training process used to deliver the content is based on evidence-based behaviour change techniques^19^ (eg, goal setting, collaborative problem-solving, positive feedback, role playing), and this process maximises the likelihood that teachers will be able to understand and apply the strategies. The intervention is solution-focused which ensures the challenges associated with implementation of the strategies are recognised and overcome.

#### Intervention materials

The intervention materials for teachers include two booklets, three sets of picture cards and 14 sets of story cards. The booklets are (1) a ‘toolbox’ book giving simple, clear guidance on how and why to use each strategy and (2) an activity book of songs, games and lesson plans. The three sets of picture cards include pictures illustrating classroom rules, friendship behaviours and emotion faces designed to facilitate the teaching of key concepts to children. Fourteen stories are depicted in story cards, and they include a range of common classroom scenarios and strategies that children can use to overcome common problems (eg, the need to share materials, wait for a turn, etc). Each set of story cards includes a discussion guide for the teacher. There are two booklets for facilitators: (1) a training manual for conducting training workshops and (2) a coaching manual for in-class support sessions.

#### Intervention delivery

The intervention is being delivered to schools randomised to intervention in the 2015/2016 academic year. Four intervention facilitators are delivering the intervention through five full-day workshops, monthly in-class coaching and fortnightly text messaging. The in-class support is delivered during one teaching session (approximately 1 h) per month in each intervention classroom and involves coaching the teacher in selected strategies, performance feedback and collaborative problem-solving. The in-class support is designed to (1) increase teachers’ confidence and motivation in using the strategies, (2) help teachers to problem-solve and (3) help tailor the strategies to the individual's classroom context. The fortnightly text messages are used to remind and encourage teachers. Each class is provided with a small amount of educational materials (eg, building blocks, books) to facilitate their use of the strategies taught.

#### Control condition

Teachers in control schools receive the same educational materials at the same time as intervention schools, and they attend the regular teacher training days provided by the Ministry of Education and Early Childhood Commission. They do not receive the Irie Classroom Toolbox training workshops, in-class support, text messages and intervention materials. Control teachers will be trained in the intervention in the 2017/2018 academic year.

### Outcome measures

All measures are collected at baseline (in the summer term of 2015) and will be repeated at post-test (summer term of 2016). All classroom and teacher-level outcomes will also be collected at 1-year follow-up (summer term of 2017). Classroom and teacher-level outcomes will be collected in the control schools only in the summer term of 2018.

All data are collected by research assistants using pencil and paper methods. Data collectors will be trained over a minimum of a 4-week period before each round of measurements. Inter-rater reliabilities will be assessed for all measures, and values of over r>0.8 will be required. Training will continue until this standard is attained. Ongoing inter-rater reliabilities will be conducted on a minimum of 5% of all measurements. Observers and testers will be rotated across classrooms and schools to ensure any variance among data collectors is not systematic. Data will be monitored weekly for such systematic variation and to ensure inter-rater reliabilities >0.8 are maintained. All questionnaire and test data will be checked by the trial manager on a daily basis for missing or erroneous data, and data will be checked weekly by a content expert to monitor compliance with the data collection protocols.

#### Primary outcomes

We have two primary outcome measures to allow us to evaluate the effect of the intervention on violence prevention at the level of (1) the teacher and (2) the children. Both measures involve observations by independent observers blind to the study design, hypothesis and group status.

Observations of teachers’ use of violence against children. These take place over two school days. Observations are continuous over one school day and for five 20 min intervals spread over the second school day linked to the schedule for class-wide observations (see below). Event recording will be used to record each use of corporal punishment, verbal abuse, and other abuse (eg, intimidation) by teachers. These behaviours are defined in an observation manual based on definitions used in Jamaican preschool classrooms previously.^5^ Teachers’ use of violence will be coded as a binary variable for analysis, with teachers coded as 1 if they use violence versus 0 if they do not. If the percentage of teachers using violence is more prevalent than anticipated and/or has wide variability in scores, then the variable will be coded as a count using three to four categories to represent the spread of the data.

Observations of class-wide child aggression. These take place over five 20 min intervals spread over one school day, and is based on a rating scale used previously in Jamaican preschool classrooms.^5^ At the end of each 20 min interval, observers rate the overall level of children's aggression in the classroom on a seven-point rating scale. The mean score over the five observations will be used in the analysis. Ratings of classroom aggression during the early childhood years predict growth in child aggression over time.^20^

#### Secondary outcomes

In addition to targeting violence prevention, the intervention also aims to promote child and teacher mental health, improve children's self-regulation skills, and improve the quality of the early childhood classroom environment. We have also previously found benefits to children's school attendance. We have secondary outcomes relevant to these aims at the level of the teacher, the classroom and the child. All measures will be conducted by researchers blind to study design, and all questionnaires will be administered in a face-to-face interview.

Classroom level measures. These include independent observations of the class-wide level of children's prosocial skills and of the quality of the classroom environment. Observations will take place over five 20 min intervals concurrently with the observations of class-wide aggression. At the end of each 20 min interval, observers will rate the level of children's prosocial behaviour on a seven-point scale, adapted from one used previously in Jamaican basic school classrooms.^5^ In addition, observers will rate the 10 seven-point scales of the Classroom Assessment Scoring System (CLASS).^21^ The CLASS tool produces summary scores of the quality of the classroom environment across three domains (emotional support, classroom organisation and instructional support). The mean scores over the five observations for each of the four outcomes (prosocial behaviour and three CLASS summary scores) will be used in the analysis. The CLASS predicts young children's social and academic skills.^22^

Teacher-level measures. Teachers will self-report on frequency of depressive symptoms using the Centre for Epidemiological Studies Depression Scale,^23^ teaching self-efficacy using four subscales from Bandura's Teaching Self-Efficacy Scale,^24^ and teacher burn-out using the Teacher Burnout scale.^25^ Strengthening the mental health of teachers of young children is one mechanism to reduce the frequency of dysregulated interactions between teachers and children.^26^

Child-level measures. Child mental health will be measured using the teacher version of the Strengths and Difficulties Questionnaire (SDQ).^27^ The total difficulties score and prosocial score will be used in the analysis.

Child inhibitory control will be measured through direct testing. Inhibitory control is a central component of self-regulation, and represents the ability to suppress impulsive thoughts or behaviours and resist distractions. Self-regulation is important for children's functioning in varied contexts, including classrooms,^28^ and self-regulation in childhood predicts criminal offending, wealth and educational achievement in adulthood,^29^ and hence, represents a key mediator for the effect of intervention on later outcomes.

The three play tests include silly sounds stroop, big/little stroop and bear/frog test. Silly sounds stroop.^30^ Children are presented with pictures of a cat and a dog and told that in the silly sounds game, dogs make the sounds of cats and vice versa. They are then presented with 18 pictures of dogs and cats and asked what sound the animal would make in the silly sounds game.

Big/little stroop.^31^ This test is adapted from the Day-Night Stroop test. Children are presented with pictures of big cats and little cats and are told to say ‘little’ when the tester points to the big cat and ‘big’ when the tester points to the little cat.

Bear/frog test. This test is adapted from the ‘bear/dragon test’,^32^ and involves performing the actions suggested by bear, and inhibiting the actions suggested by frog.

A composite variable of inhibitory control will be created by summing the standardised score of these three tests.

Child attendance will be recorded from school records.

#### Participant timeline

**Figure 1:**
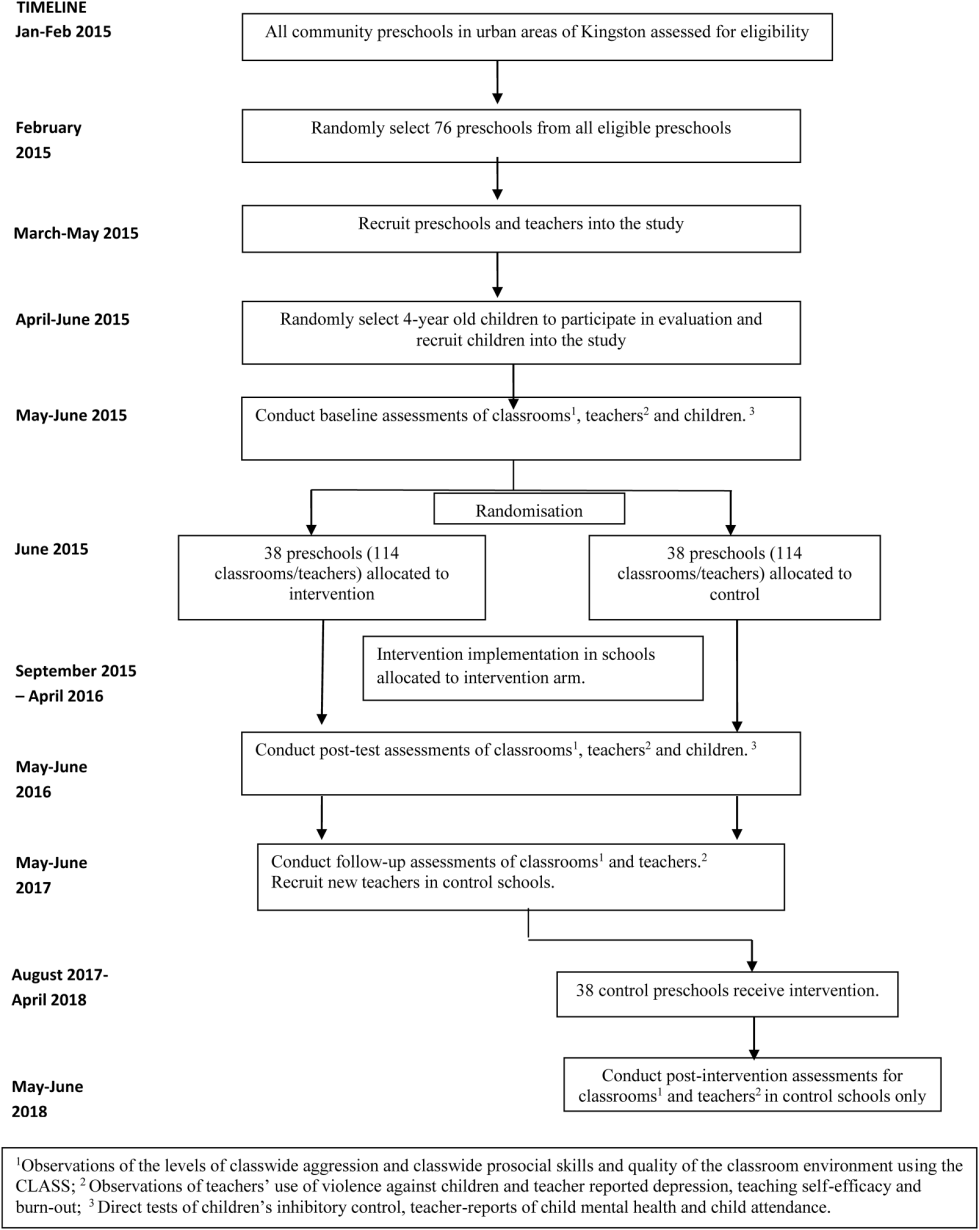
Trial timeline.

The trial timeline is show in figure 1. The trial is a 4-year study. Initial recruitment and baseline assessments take place in the summer term of the first year of the study. The intervention is delivered to the intervention schools throughout the second year of the study, and post-test measures are collected in the summer term of that year. In the third year of the study, 1-year follow-up measurements will be collected in all schools. The comparison schools receive the intervention in the final year of the study with assessments of teachers’ use of violence, level of class-wide child aggression and prosocial skills, the quality of the classroom environment and teacher depression, self-efficacy and burn-out at the end of the year.

### Sample size

The trial has two primary outcomes: level of class-wide aggression and violence against children. Using Tukey's method of correction for multiple testing,^33^ we require a significance level of 0.036 in each individual test to keep a Family-Wise Error Rate of 0.05.

Level of class-wide aggression. In a parallel group design, 72 classes in each group are sufficient to detect an ES of 0.5 SD, with 81% power at 0.05 level of significance. With a cluster size of three (3 classrooms/school), and assuming an intraclass correlation (ICC) of 0.25, the design effect is 1.5 (design effect=1+(cluster size–1)×ICC)) giving a required sample size of 108 classrooms per group, which yields 36 schools per group. We will recruit 38 schools (114 classrooms) per group (76 schools in total) to allow for 9% teacher loss.

Violence against children by teachers. In a parallel group design, 90 classes in each group are sufficient to detect a reduction from 60% to 35% with 87% power at 0.05 level of significance. With a cluster size of three (3 classrooms/school) and an ICC of 0.1 (based on data from our efficacy trial), the design effect is 1.2, giving a required sample size of 108 classrooms per group, which yields 36 schools in each group. As mentioned above, we will recruit two additional schools per group.

The analyses of the secondary outcomes is considered exploratory, and the significance level of the tests will not be adjusted for comparisons of multiple outcome.

Classroom and teacher-level measures. Similar to the primary outcome of level of classwide aggressions, we have 85% power at a 0.05 level of significance to detect a difference of 0.5 SD on the quality of the classroom environment and on teacher depression, self-efficacy and burn-out.

Child-level measures. In a parallel group design, 252 children in each group are required to detect a difference of 0.25 SD with 80% power at 0.05 level of significance. With 11 children per school, and an estimated intracluster correlation coefficient of 0.05, the design effect is 1.50 giving a required sample size of 378 children and 36 schools in each group. Our proposed sample includes 38 schools/group. We recruited children from the 4-year-old class: in classes with 12 or fewer children, all children were recruited; where there were more than 12 children, a random sample of 12 children were selected. The maximum sample size was 456 children/group.

#### Allocation

Schools were randomised to intervention or control using a computer-generated simple-randomisation sequence by an independent statistician who was masked to school identity. All schools and children were recruited prior to randomisation. All baseline measurements were conducted prior to randomisaton.

#### Blinding

Researchers will be masked to intervention group at post-test. To maintain masking of researchers, observers and interviewers will not be informed of the study design, and will be employed during the measurement phases of the study only. Intervention and control schools will be provided with the same educational materials. Teachers will be asked not to reveal their school's intervention status to data collectors. Where research assistants collect data at more than one timepoint (eg, baseline and post-test), they will be rotated across schools and classrooms, so they do not observe the same classroom from one timepoint to the next, and hence, are not able to detect changes to teacher competencies.

#### Data management

Data will be double-entered into OpenClinca V.3.1 by two independent data entry personnel. The OpenClinica database includes a series of logical data checks, with errors generating queries. The status of all data queries will be maintained in an automated query system which forms part of the OpenClinica installation. Sign-off (electronic signature) will be required at each review resolution and at the finalisation of the data series for each participant. Trial-related electronic information will be stored on a dedicated data server with restricted and documented human access. Automated daily backups are performed to a distinct data server, and weekly offsite backups to ensure a geographically separated data copy. Hard copy questionnaires will be scanned, with scanned copies stored in a secure document repository (AlFresco software) on the same data server and subject to the same backup regime. Hard copy questionnaires will be stored in a secure physical location with restricted human access, and with any participant identifiers kept separately from trial data.

#### Statistical methods

The effect of intervention will be examined on an intention-to-treat basis, using multilevel multiple regression models for continuous outcomes and random effects logistical regression models for binary outcomes (and random effect for count outcomes such as Poisson or Negative Binomial if required), to take into account the hierarchical structure of the study. Tester/observer and intervention status (and child age and sex for child-level data) will be entered as fixed effects, and school (and classroom) entered as random effects.

Few studies have reported on mediators of intervention effectiveness in school-based preventative trials. We will explore whether change in teacher depression, self-efficacy and burn-out mediate the effect of intervention on teachers’ use of violence and levels of classroom aggression using standard techniques for multilevel mediation analyses. We will also examine whether teachers’ use of violence and the quality of the classroom environment mediate the effect of intervention on child mental health, self-regulation and attendance. Potential moderators of intervention effectiveness will also be investigated. We will examine whether teachers who use high levels of violence against children at baseline, and whether classrooms characterised by high levels of child aggression at baseline, benefit most from the intervention. We will also examine whether the child's sex moderates the effect of intervention on child mental health, social skills, self-regulation and school attendance.

#### Economic analysis

The goal of the economic analysis is twofold. First, we will assess the efficiency of classroom instruction (using observational data of the quality of the classroom environment and the level of childhood aggression). As a short-term indicator of the effectiveness, we will compare the costs of training with time saved in instruction attributed to reduced behavioural disturbances. This can be coupled with the data on benefits to child attendance (proportional to the total days so that a 5% increase of attendance is worth 5% of what a year of schooling is worth) to assess changes in classroom efficiency. We will link the observed changes in attendance and child self-regulation skills with data from other studies on preschool instruction, to provide a plausible range of expected long-term economic returns. By assessing the degree to which the changes in teacher behaviour are present a year after the interventions we will be able to assess whether the initial training costs need to be augmented with refresher training.

## Ethics and dissemination

School principals were visited by a senior member of the research team and were informed of the study and invited to participate. Once the principal had agreed, the teachers were approached individually by the same personnel, and informed consent was sought. Consent forms were sent home to parents of children selected to participate in the evaluation, and returned to the school.

Ethical consent for the study was given by the School of Psychology Ethics and Research Committee, Bangor University, on 29 October 2014 (ref: 2014-14167), and by the University of the West Indies Ethics Committee on 14/15 February 2015 (ref: ECP 50, 14/15).

The results of the study will be released to the participating schools and teachers, the Ministry of Education, the Early Childhood Commission, and teacher training institutions in Jamaica, and will be submitted to peer-reviewed journals regardless of the magnitude or direction of the effects.

## Discussion

There is significant evidence that early childhood education interventions can promote young children's development in LMIC,^34^ and many countries are investing in preschool provision; however, provision of high-quality preschool remains a challenge. Early childhood is a particularly sensitive period, as experiences in early childhood have long-term effects on brain function, cognition and psychosocial functioning. A safe (free from physical and psychological harm), secure (consistent and predictable), and nurturing (sensitive and responsive) early childhood caregiving environment promotes child physical and mental health over the long term.^35^ There is limited evidence of the effectiveness of interventions aimed at improving the quality of preschools in LMIC, although several small-scale studies have shown promising results to teacher practices and children's mental health.^36^ Through our previous work in Jamaica, we have shown that training teachers in evidence-based behaviour management strategies can reduce violence against children by teachers and reduce conduct problems and increase social skills in antisocial young children.^12^ Through this study, we will test whether a simplified and lower cost intervention, implemented on a larger scale will lead to meaningful benefits to teachers and children. If this intervention were effective at improving the caregiving environment of young children in school this would have significant implications for the prevention of child mental health problems in LMIC where services are often limited. As the intervention is integrated into the school system and involves training existing staff to improve their performance (rather than to undertake new duties), it represents an appropriate strategy for large-scale implementation.

### Trial status

The study began in January 2015. Baseline data collection was conducted from May to June 2015. Intervention began in September and is currently underway.
